# Post kala-azar dermal leishmaniasis: an unresolved mystery

**DOI:** 10.1016/j.pt.2013.12.004

**Published:** 2014-02

**Authors:** Debanjan Mukhopadhyay, Jane E. Dalton, Paul M. Kaye, Mitali Chatterjee

**Affiliations:** 1 Department of Pharmacology, Institute of Postgraduate Medical Education and Research, 244 B, Acharya JC Bose Road, Kolkata 700 020, India; 2 Centre for Immunology and Infection, Hull York Medical School and Department of Biology, University of York, Wentworth Way, York, YO10 5DD, UK

**Keywords:** antimony, post kala-azar dermal leishmaniasis (PKDL), UV light, vitamin D, regulatory T cells

## Abstract

Post kala-azar dermal leishmaniasis (PKDL), a cutaneous sequela of visceral leishmaniasis (VL), develops in some patients alongside but more commonly after apparent cure from VL. In view of the pivotal role of PKDL patients in the transmission of VL, here we review clinical, epidemiological, parasitological, and immunological perspectives of this disease, focusing on five hypotheses to explain the development of PKDL: (i) the role of antimonial drugs; (ii) UV-induced skin damage; (iii) reinfection; (iv) organ specific failure of memory T cell responses; and (v) genetic susceptibility of the host. This review will enable researchers and clinicians to explore the unresolved mystery of PKDL and provide a framework for future application of ‘omic’ approaches for the control and eventual elimination of VL.

## Highlights

• Sodium antimony gluconate contributes towards the pathogenesis of PKDL.
• UV light plays a pivotal role in the development of PKDL.
• Development of PKDL can be viewed as a reinfection or activation of latent *Leishmania* parasites.
• PKDL can be resolved by mounting an effective tissue-specific memory T cell response.
• Host genetic factors play a contributory role.

## PKDL: current scenario

The leishmaniases comprise a diverse group of poverty-related neglected tropical diseases that have a major impact on health worldwide. Among the manifestations of leishmaniasis, post kala-azar dermal leishmaniasis (PKDL; see Glossary) caused by the protozoan parasite *Leishmania donovani*, is possibly the most intriguing clinically and scientifically, as it generally develops as a sequela after apparent successful cure from visceral leishmaniasis (VL; also known as kala-azar) 1, 2. The clinical presentation of VL and PKDL differ substantially; in VL, patients suffer from prolonged fever, hepatosplenomegaly, weight loss, and anaemia, whereas manifestations of PKDL are limited to macular, papular, or nodular lesions in the skin [2]. PKDL is confined to two geographically distinct zones, namely South Asia (India, Nepal, and Bangladesh) and East Africa, mainly Sudan 2, 3. In the South Asian variant, polymorphic lesions (coexistence of macules/patches along with papulonodules) are prevalent, whereas the Sudanese variant has papular or nodular lesions. Although mortality from PKDL is low, it is a stigmatizing disease that carries a significant socioeconomic burden, further amplified by a reluctance to obtain treatment or due to noncompliance. Lesions, especially the papulonodules, are parasite-rich, driving speculation that PKDL plays a pivotal role in the inter-epidemic transmission of VL.

**Figure 1:**
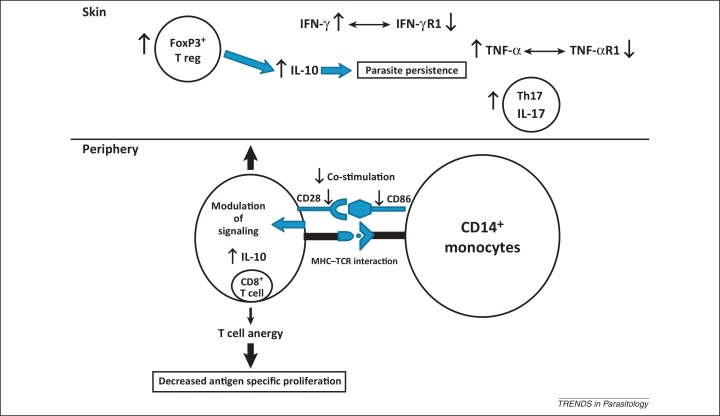
Current immunological scenario in Indian PKDL. Patients with Indian PKDL have distinct patterns of immunity in the skin and periphery. In the skin, immunity is regulated by IL-10 and FoxP3 9, 43 because despite the enhanced levels of IFN-γ and TNF-α, their respective receptors are downregulated 37, 74. Additionally, in the skin, there is an increased presence of Th17 cells and IL-17 [75]. By contrast, peripheral immunity is controlled mostly by CD8^+^ T cells that are the major sources of IL-10 and are anergic in nature 2, 9. Lack of co-stimulation was also evident because decreased CD28 and CD86 was found in circulating CD8^+^ T cells and CD14^+^ monocytes, respectively 9, 21. Abbreviations: PKDL, post kala-azar dermal leishmaniasis; IL-10, interleukin 10; FoxP3, forkhead box P3; IFN-γ, interferon γ; TNF-α, tumour necrosis factor α; Th, T helper.

Understanding the clinico-epidemiological aspects of PKDL would help define strategies for controlling VL, by providing further insights into *L. donovani* transmission dynamics. Most epidemiological studies with PKDL have reported no gender bias 4, 5, 6, 7, 8, a notable exception being studies from our group where a male predominance was reported [9]. The age distribution of PKDL in South Asia and Sudan also differs, as in the former, young adults are more affected whereas in the latter, children are more affected [4]. A lag period ranging from 2 to 10 years exists between cure from VL and onset of PKDL, suggesting that PKDL echoes the epidemic of VL and can persist well after the epidemic. This is supported by an epidemiological study in Bangladesh where it was noted that the incidence of PKDL showed a steep rise from 1 case per 10 000 in 2002–2004 to 21 cases per 10 000 in 2007 [6]. In India, the incidence of VL peaked in 1992 and a smaller rise was reported in 2007 (http://www.who.int/leishmaniasis/resources/INDIA.pdf). Therefore, extrapolating from the Bangladesh experience, the incidence of PKDL in India may well see a sharp rise in coming years. In South Asia, transmission of VL is anthroponotic, whereas in Sudan, it is zoonotic and anthroponotic; therefore, patients with PKDL are the proposed disease reservoir of VL in India [10]. Accordingly, eradication of PKDL should be an essential component of the current VL elimination programme in South Asia that aims to bring down the annual incidence of VL to less than 1 per 10 000 population at a district or sub-district level by the end of 2015 (http://www.who.int/tdr/publications/documents/kala_azar_indicators.pdf). To achieve this goal, a greater understanding of the cause(s) of PKDL is essential. Many excellent reviews have covered the epidemiology, immunopathology, diagnosis, and treatment of PKDL; Box 1 and Figure 1 summarise our current knowledge of the immune responses observed in PKDL 2, 4, 11, 12, 13, 14. Information regarding the aetiopathogenesis of PKDL is limited and, therefore, no consensus has emerged to explain what factors modify the behaviour of the normally viscerotropic *L. donovani* parasite to become dermatotropic and manifest as PKDL. In the following sections, we review key mechanisms suggested to be involved in the development of PKDL.

Box 1 **Immunology of PKDL**

The precise immune mechanisms of PKDL are still obscure and, interestingly, the immunobiology of the Sudanese and South Asian PKDL differ. Therefore, information from one is not extrapolatable to the other [2]. In Sudanese PKDL, because of the shorter time lag between cure from VL and development of PKDL, the disease-associated immune involvement mimics the scenario of immune reactivation after cure from VL. PBMCs from Sudanese PKDL patients react and proliferate following induction by *Leishmania* antigens and secrete more IFN-γ, whereas IL-10 was produced primarily from CD4^+^ T cells [52]. By contrast, South Asian PKDL is more chronic due to the longer gap between cure from VL and disease onset. Here, CD8^+^ T cells predominated in lesions and circulation 53, 73. Tregs also play an important role in the lesional immunology of South Asian PKDL, as evident by their elevated mRNA expression of FoxP3, CTLA-4, and CD25 9, 43. In addition to lesional immunology, systemic immune changes include increased antigen-induced IL-10 within circulating CD8^+^ T cells and impairment of antigen-induced proliferation. These cells were anergic in nature because they lost their surface co-stimulatory CD28 molecule 9, 73. Furthermore, the interacting partner of CD28, known as B7.1 or CD86, on monocytes was decreased, suggesting that an immunosuppressive milieu occurs in circulation [21]. Lesional immunology of PKDL was similar in South Asian and Sudanese PKDL with enhanced expression of IL-10, TGF-β, IFN-γ, and TNF-α. However, despite the higher levels of IFN-γ and TNF-α, the expression of IFN-γR and TNFR1 was lower in patients with PKDL in India and increased after treatment 37, 74. Similarly, in the Sudanese variant, a genetic polymorphism was found in IFN-γR [63]. In Sudanese PKDL, expression of IL-10 from keratinocytes was considered as a key factor and a predictor for development of PKDL, particularly after cure from VL [4]. Moreover, the decreased presence of E-LCs plays a contributory role for immune suppression. Recently, elevated levels of IL-17, its transcription factor ROR-γt, and IL-22 in lesions and circulation (plasma and lymphocytes) were reported [75]. Taken together, immunological studies conducted so far indicate that PKDL is not a localised disease, but involves systemic immunity. Figure 1 in main text summarises our current knowledge of the local immune response in patients with PKDL.

## A role for antimonial drugs

Epidemiological data and clinical reports have strongly suggested a link between administration of sodium antimony gluconate (SAG) and subsequent development of PKDL [15]. In India, 62 out of 85 (73%) patients with PKDL who were followed up for 9 years after cure from VL were treated with SAG for VL [8]. A minority (23/85, 27%) developed PKDL after being treated for VL with amphotericin B (*n* = 13), ambisome (*n* = 2), miltefosine (*n* = 2), miltefosine–amphotericin B (*n* = 1), or paromomycin (*n* = 5), respectively. In Sudan, Bangladesh, and Nepal, 100% of PKDL patients received SAG 4, 5, 6, 7, suggesting that SAG could well influence the development of PKDL. A small subgroup of 15–20% of patients with PKDL gave no prior history of VL, attributable to subclinical infection and had therefore not received any drug during that period [8]. It is interesting, however, that SAG can and is still being used for the treatment of PKDL, although at a higher dose and for a prolonged period (20 mg/kg/day, intramuscular, for 4 months) than required for the treatment of VL (20 mg/kg/day for 3 weeks). It is also plausible that differences in systemic versus skin concentrations of antileishmanial drugs permit the survival of parasites in the latter. However, before incriminating SAG for the development of PKDL, further data are required on the frequency of PKDL in cured VL patients treated with other more recently introduced antileishmanial drug regimens. Due to the rise in antimony resistance, there is now a long history of using miltefosine and amphotericin B to treat patients with VL in India [16]. As noted above, Thakur *et al.* reported that using amphotericin B (20 mg/kg) for the treatment of VL effectively minimised development of PKDL, whereas in individuals treated with lower doses of amphotericin B (15 mg/kg), PKDL was reported [10]. Patients who received miltefosine, paromomycin, and a combination therapy of amphotericin B/miltefosine have rarely been found to develop PKDL 17, 18, 19. Collectively, available data strengthened the notion that SAG directly or indirectly influences the incidence of PKDL, but definitive evidence may require another decade, because in India, PKDL can develop 20–40 years after cure from VL 2, 18. Another speculation is that antileishmanial drugs used at lower doses and for a shorter duration eliminated parasites from the viscera, not from the skin, which required a higher dose. Hence, PKDL might not only be drug-related but could also result from a dose-related phenomenon (Hypothesis 1). A more formal understanding of drug pharmacokinetics/pharmacodynamics is clearly warranted.

In addition to epidemiological evidence, immunological data also support this hypothesis, because levels of immunoregulatory cytokines transforming growth factor β (TGF-β) and interleukin 10 (IL-10), factors that support parasite persistence, remained high even after completion of treatment with SAG [20], whereas this was not the case with amphotericin B or miltefosine 20, 21. Furthermore, in an *in vitro* model, treatment of THP1 macrophages with SAG caused elevation of two anti-inflammatory, disease-sustaining molecules, namely haem oxygenase 1 (HO-1) and glutathione [22]. Another contributory factor is the inability of SAG to restore the peroxisome function of the host [23]. Additionally, the failure of SAG to provide a sterile cure strongly indicates the possibility of drug-induced genetic alterations in resistant parasites; SAG-resistant parasites are proposed to have an enhanced degree of ‘fitness’ [24]. Another pertinent observation is that PKDL-causing strains expressed higher levels of promastigote surface antigen (PSA2) and glycoprotein 63 (gp63), molecules associated with dermatotropism; concomitantly, they had decreased expression of amastigote antigen 2 (A2), which is linked with enhanced viscerotropism 25, 26. Given our recent understanding of the plasticity of the *Leishmania* genome [27], and improved capacity and cost reductions associated with parasite genome sequencing, future studies aimed at characterising PKDL isolates with various drug histories are now clearly essential and have become technically and financially achievable.

## UV light contributes towards development of PKDL

**Figure 2:**
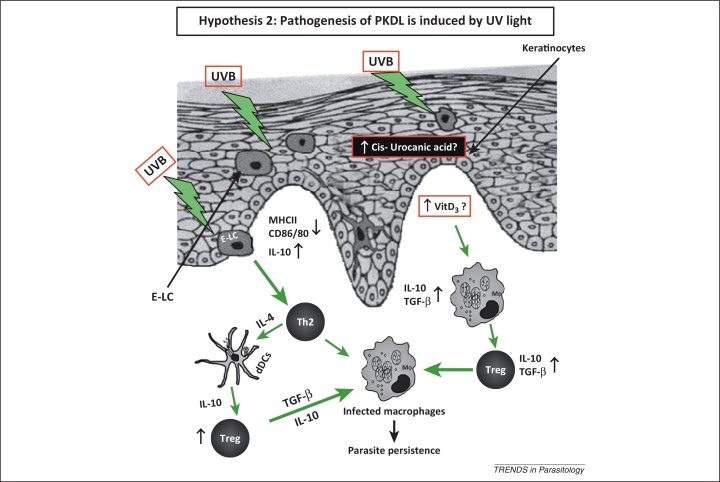
Pathogenesis induced by UV light. UV light has a potential role in the pathogenesis of PKDL via enhanced secretion of *cis*-urocanic acid from keratinocytes and vitamin D_3_. This translates into a reduced number of E-LCs, which also have an altered morphology [38]. In E-LCs, expression of MHCII, CD80, and CD86 are decreased, and IL-10 is increased 9, 38. IL-4 secreted from Th2 cells subsequently activates dDCs to secrete more IL-10, which then induces an increased presence of Tregs, allowing for parasite persistence. Similarly, TGF-β from infected macrophages can activate Tregs, which then produce more TGF-β, facilitating parasite persistence. Another possibility is that raised levels of vitamin D_3_ polarise macrophages towards an alternatively activated phenotype that along with an enhanced presence of Tregs collectively generates an immunosuppressive milieu. Abbreviations: PKDL, post kala-azar dermal leishmaniasis; dDC, dermal dendritic cell; E-LC, epidermal Langerhans cells; MHC, major histocompatibility complex; IL-4, interleukin 4; IL-10, interleukin 10; Treg, T regulatory cell; TGF-β, tumour growth factor β; Th, T helper.

Lesions of PKDL consistently appear on sun-exposed areas, particularly the face, ears, arms, etc., rather than unexposed areas, such as the scalp and chest. This supports the concept that exposure to UV light plays a contributory role in the pathogenesis of PKDL (Hypothesis 2) (Figure 2) [28]. This role of UV light is further highlighted by the characteristic presence of photosensitivity in patients with PKDL. The potent immunosuppressive property of UV light is linked to its ability to damage the antigen presenting epidermal Langerhans cells (E-LCs) and inhibit contact hypersensitivity and alloantigen responses [29]. UVB light (280–320 nm)-induced immunosuppression can operate either through its chromophore *cis*-urocanic acid or via modulation of vitamin D_3_ (Figure 2) 30, 31. During keratinisation, *trans*-urocanic acid produced from histidine undergoes *trans* to *cis* UV-induced photoisomerisation. All dermal cell types are affected, especially E-LCs, wherein their numbers are reduced and their morphology, particularly, the characteristic dendritic pattern is altered; together, this attenuates the antigen presenting property of dermal cells 32, 33. These E-LCs from UV-irradiated skin have reduced expression of major histocompatibility complex (MHC) class II, along with a loss of co-stimulatory molecules, CD80 and CD86, which translates into impaired antigen presentation (Figure 2) 34, 35. Additionally, by exerting its influence on keratinocytes and lymphocytes, *cis*-urocanic acid modulates a vast array of cytokines that range from proinflammatory tumour necrosis factor α (TNF-α) to anti-inflammatory and immunosuppressive IL-10 [30]. In view of the fact that 40% of the adult population are UVB sensitive, based on their impairment of hapten-induced contact-dependent hypersensitivity, this may also account for the low proportion of individuals developing PKDL after cure from VL [28].

Studies on lesional patterns often mirror the clothing habits of individuals, which strongly suggest a link between exposure to UV light and the pathogenicity of PKDL [36]. However, no such study has been undertaken in India but, notably, there is a consistent sparing of photoprotected areas. Available data from Indian PKDL showed a downregulation of co-stimulatory molecules [21], similar to that resulting from UV irradiation [30], and an increased expression of cytokines TNF-α and IL-10 at the lesional site 37, 38. These data endorse a causal role for UV light in both disease pathogenesis and immunosuppression.

UV, vitamin D_3_, and dihydroxylated vitamin D_3_ [1α,25(OH)_2_D_3_] have a potent immunomodulatory role 31, 39 in which UV light enhances the synthesis of vitamin D_3_ (Figure 2). 7-Dehydrocholesterol absorbs UV light and is converted into vitamin D_3_ by a series of enzymatic and non-enzymatic reactions. The circulating vitamin D_3_ binds to vitamin D-binding protein (VBP) and subsequently enters immune cells, such as monocytes and macrophages. Within the cells upon further hydroxylation by a mitochondrial enzyme CYP27B1, the active 1α,25(OH)_2_D_3_ is formed, which then forms a complex with vitamin D receptor (VDR). The complex then translocates into the nucleus, and after binding to the vitamin D response elements (VDREs), induces synthesis of antibacterial peptides and immunomodulatory factors, such as cathelicidin (LL-37), TGF-β, and arginase 40, 41. The influence of vitamin D_3_ on the immune system appears dichotomous. On the one hand, it induces synthesis of potentially host-protective antibacterial peptides from macrophages, whereas on the other, it inhibits Toll-like receptor (TLR)-induced activation of macrophages, downregulates co-stimulatory molecules and proinflammatory cytokines, and also induces TGF-β and IL-1. This ability to upregulate the arginase pathway implies that 1α,25(OH)_2_D_3_ or vitamin D_3_ facilitates alternative activation of macrophages [42]. Additionally, the increased presence of regulatory T cells (Tregs) promotes IL-10 secretion, thereby suppressing the local immune response and supporting parasite persistence. In Indian PKDL, an increased presence of forkhead box P3 (FoxP3), a key molecular marker of Tregs, has been reported 9, 43. Indeed, in B6.*Vdr*^−/−^ mice (which lack the gene encoding the vitamin D receptor), resistance to *Leishmania major* infection is enhanced. Conversely, treatment of wild type mice with 1α,25(OH)_2_D_3_ led to a VDR-dependent inhibition of macrophage killing, induction of arginase, and downregulation of inducible nitric oxide synthase (iNOS) [44]. A strong case between exposure to UV and PKDL in terms of dermal phenotypic changes exists, but does not necessarily constitute an argument for causation. Nevertheless, there is sufficient data to warrant further exploration of the role of UV light in the aetiopathogenesis of PKDL, potentially feasible in animal models. Alternatively, a metabolomic analysis to determine metabolites of the vitamin D_3_ pathway could strengthen this hypothesis.

## PKDL: a disease of reinfection or parasite persistence?

A hallmark of many infectious diseases is persistence of the pathogen after clinical cure, which occurs in tuberculosis, viral infections (e.g., herpes), and protozoan diseases (e.g., trypanosomiasis) [45]. In leishmaniasis, evidence of parasite persistence after clinical cure exists 46, 47, and in areas where leishmaniasis is endemic, recurrence has been attributed to parasite persistence and/or reinfection (Hypothesis 3). The optimal approach would be to characterise these parasites and compare them with the parental type, preferably using genomic and/or proteomic analysis. This would establish whether the parasites were sourced from the visceral form of the disease or after reinfection. In a mouse model, parasites that persisted were genetically similar to the parental clone [46]; however, analogous studies in humans are logistically not possible, in view of the parasite density being insufficient for establishment of parasite isolates 45, 47. Because strains isolated from individuals with VL and PKDL in India have genetic heterogeneity [48], the axis tends to tilt in favour of reinfection. On the contrary, it can be hypothesised that parasites reside within an alternative cell type, such as fibroblasts or keratinocytes, during the latency period and, when reactivated, cause PKDL [25]. To address this issue, parasites would need to be isolated from a patient suffering from VL and compared with those isolated from the very same individual when PKDL develops, which in practical terms is very difficult owing to the long and unpredictable lag period between VL and onset of PKDL. However, such studies are feasible in Sudan where the onset of PKDL following VL is much shorter [4]. In Sudan, PKDL is probably due to parasite persistence because patients cured of VL developed PKDL even after relocating to an area where VL is not endemic (A.M. El Hassan *et al*., personal communication). Another challenge to the reinfection theory is why should genetically different strains exclusively cause PKDL, and not re-emergence of VL? An important factor possibly preventing reinfection could be that VL generates a strong systemic memory response, but a concomitant failure of tissue-specific immunity allows parasites to multiply in the skin.

## Failure of tissue-specific T cell memory

**Figure 3:**
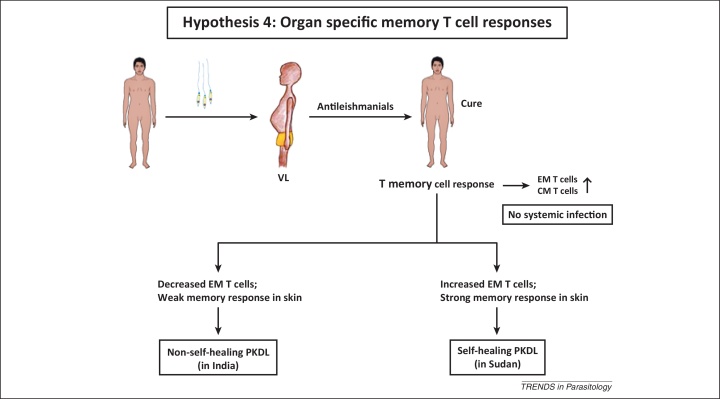
Failure of organ-specific memory T cell response. Following infection with *Leishmania* parasites, individuals develop VL, and generally antileishmanial treatment results in lifelong immunity due to the presence of systemic CM and EM T cells. In patients with self-limiting PKDL (e.g., in Sudan), an increased EM T cell response in the skin causes spontaneous resolution. However, in Indian PKDL, a weak skin-specific EM T cell response accounts for its non-self-healing nature. Abbreviations: CM T cells, central memory T cells; EM T cells, effector memory T cells; VL, visceral leishmaniasis; PKDL, post kala-azar dermal leishmaniasis.

Recovery from *Leishmania* infection is often associated with development of a strong memory immune response that in humans confers lifelong protection against reinfection (Figure 3) [49]. However, under certain conditions, such as immunosuppression, this infection-induced immunity may be impaired, rendering the host susceptible to infection and/or reactivation of latent parasites [50]. The diverse tissue microenvironments where parasites are found in VL, such as the liver and spleen, qualitatively and quantitatively influence various aspects of host immunity [51]. In this context, PKDL poses an interesting challenge because despite acquisition of systemic protective immunity, apparent from the recall cytokine or proliferative T cell responses measured in peripheral blood mononuclear cells (PBMCs) or whole blood assays 9, 52, organ-specific immune deficits do occur in the skin. This was evidenced by a local increase of Tregs expressing the transcription factor FoxP3 and a preponderance of CD8^+^ T cells 9, 53 (Hypothesis 4, Figure 3).

PKDL in Sudan and South Asia differ significantly in terms of their disease outcome: in Sudanese PKDL, the lesions regress spontaneously, whereas in South Asia, prolonged treatment is essential [4]. This difference might be correlated with the lag period of the disease, because in Sudan patients develop PKDL with a much shorter lag period or immediately after cure from VL and their immunological responses might mimic post-VL features, that is, active immune memory. In South Asia, there is a long lag period and immunological responses differ significantly and show immune anergy, that is, failure to elicit an antigen-specific immune response [9].

Immune memory is a property of central memory (CM) T cells (having a CD62L^hi^, CD45RB^hi^, and CCR7^hi^ phenotype) and effector memory (EM) T cells (having a CD62L^low^, CD45RB^hi–low^, and CCR7^low^ phenotype) [49], the latter being more functionally relevant than the former [54]. However, in Indian PKDL due to the paucity of T helper (Th) cells in the dermal lesions [53], the disease acquires a non-self-healing nature. However, in Sudan, an increased dermal presence of CD4^+^ T cells is associated with self-healing PKDL. This concept would hold if comparative studies with the non-self-resolving Indian and self-healing Sudanese PKDL showed different memory responses. In Indian PKDL, antigen-specific recall responses as measured by response to *Leishmania* antigen or phytohaemagglutinin (PHA) stimulation in circulating CD4^+^ T cells was intact, whereas CD8^+^ T cells were functionally impaired [9]. Therefore, establishing the functional ability of these CD8^+^ memory T cells is necessary. Another important factor that might play an important role in the distinct pathology of Indian and Sudanese PKDL is the expression of CD25 (the IL-2 receptor), present on the regulatory T cell surface [43]. In Sudanese PKDL, dermal biopsies showed a decreased presence of CD25^+^ T cells [38], whereas in the Indian variant, cells bearing CD25 were significantly increased [43].

For homing of memory CD4^+^ T cells, increased expression of the cutaneous lymphocyte antigen (CLA) is important for self-resolution as observed in localised self-resolving cutaneous leishmaniasis (CL), whereas the lack of CLA expression in diffuse CL (DCL) correlates with a non-healing outcome [55]. One can therefore envisage that in PKDL, aberrant expression of CLA prevents CD4^+^ EM T cells from homing to the dermal lesions. Granuloma formation in the skin has been proposed to confer protection against leishmaniasis as evidenced in CL [56]. Importantly, in PKDL, granuloma formation is scanty and because Indian PKDL is accompanied with a substantial decrease in the proportion of CD4^+^ T cells 38, 52, 53, 57, it strengthens the notion that impaired tissue-specific immunity underpins disease persistence.

## Host genetic susceptibility factors

**Figure 4:**
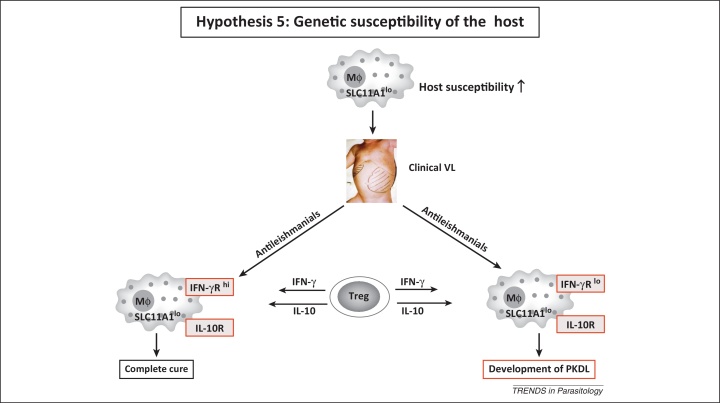
Genetic susceptibility of the host. A model indicating the possible interplay of genes that contributes towards host susceptibility in PKDL. SLC11A1 enhances macrophage activation in terms of its antimicrobial activity (e.g., iron sequestration from parasites) [72]. Polymorphisms of *SLC11A1* (*SLC11A1*^lo^) can render the host susceptible to VL; additionally, in these individuals, a polymorphism in the *IFN-γR* gene (IFN-γR^lo^) makes them more susceptible to developing PKDL, owing to the reduced functionality of the IFN-γR gene. Tregs secrete both IL-10 and IFN-γ, but due to the decreased activity of IFN-γR, the immune deactivating properties of IL-10 predominate; conversely, IFN-γR^hi^ individuals are cured of VL and are less prone to develop PKDL. Abbreviations: Mϕ, macrophage; SLC11A1, solute carrier family 11A1; IFN-γR, interferon γ receptor; IL-10R, interleukin 10 receptor; Treg, T regulatory cell; VL, visceral leishmaniasis; PKDL, post kala-azar dermal leishmaniasis.

All the factors discussed above have focused on different aspects of host immunity, wound healing, and regeneration of the immune memory response, all of which can potentially be regulated by the host genotype. Various forms of leishmaniasis have been subjected to intense genetic analysis in murine models and humans, but data pertaining to patients with PKDL remain preliminary and are restricted to three studies 58, 59, 60, 61. However, because all three studies were performed in Sudan, the conundrum remains unresolved as to why some but not all patients with VL develop PKDL. A significant association between interferon γ receptor (IFN-γR polymorphism) and development of PKDL has been reported (Hypothesis 5, Figure 4) 62, 63. This was important because several studies have reported an increased presence of both proinflammatory and anti-inflammatory cytokines in the lesions 9, 37, 52. Polymorphisms in IFN-γR are associated with its decreased functionality and expression that resulted in decreased responsiveness, despite high levels of IFN-γ. Although a critical balance between IFN-γ versus IL-4 and/or IL-10 secreting cells is essential in leishmaniasis [64], no correlation was found between IFN-γ or IL-10 promoter polymorphism and disease susceptibility 63, 65. Furthermore, the failure to respond adequately to IFN-γ during active disease due to IFN-γR1 being compromised would facilitate parasite growth and multiplication. Indeed, expression of IFN-γR1 is lower in individuals with Indian PKDL as compared with healthy controls [37].

In Sudan, PKDL tends to develop in patients with VL having higher levels of plasma C-reactive protein (CRP), but the question remains as to whether genetic polymorphisms in CRP genes or their promoters correlate with individuals having a higher propensity to develop PKDL [66]. Genes involved in innate immunity, particularly those associated with complement activation, such as *FCN*-2 (encoding Ficolin-2), are associated with an enhanced susceptibility to CL 67, 68. Polymorphisms at the promoter regions of the *FCN*-2 gene are associated with its decreased expression and increased susceptibility to CL [68]. The increased expression of mannose-binding lectin 2 (MBL2) was stated to be associated with VL and, indeed, increased serum levels of MBL2 and genetic polymorphism at the promoter and exons of *MBL2* correlated with disease susceptibility [69]. Another important candidate gene for leishmaniasis is *SLC11A1* (formerly known as *NRAMP1*), which encodes the solute carrier family 11a (SLC11A1). SLC11A1 functions as a proton/divalent cation antiporter present on the endosomal membrane of phagocytes. SLC11A1 has diverse effects on macrophage activation, including regulation of IL-1β, iNOS, MHC class II molecules, TNF-α, nitric oxide (NO) release, oxidative burst, and antimicrobial activity [70]. Polymorphisms of *SLC11A1* at the promoter and exons make it functionally null and are associated with increased susceptibility to VL (Figure 4) 71, 72. The lack of studies with PKDL emphasises the need to establish susceptibility determinants. Additionally, it can be envisaged that investigation of epigenetic modification(s) in the aforementioned genes would help establish the underlying cause(s) for development of PKDL.

## Concluding remarks

PKDL is a perplexing disease, in particular for the shift in parasite tropism after the onset of apparent cure from VL and for the geographical variation in clinical presentation. The factors discussed above provide suitable explanations for PKDL, at least in some geographical settings. The lack of mouse models of PKDL precludes formal testing of some of these hypotheses but they have helped focus attention on how best to analyse the available clinical material. Thus, it can be concluded that development of PKDL is multifactorial and one/more of the hypotheses discussed above have a contributory role on the development of the disease, and future research should aim to address the several unanswered yet relevant questions (Box 2). Studies of parasite genotypes and phenotypes are clearly a high priority and need to be intimately linked to state-of-the-art functional histopathology, including, for example, tissue-based transcriptomics. In years to come, creative clinical studies on this challenging disease merged with the application of the latest ‘omics’ approaches are sure to yield new insights into the complexity of the *Leishmania*–host interaction and open new doors for disease control.

Box 2 **Outstanding questions**

• Does treatment with sodium antimony gluconate cause any epigenetic modifications of the host or parasites?
• Can any secondary skin infection lead to activation of latent parasites?
• Can host nutritional factors such as trace elements influence the strong systemic immunity in patients with PKDL?
• Are host/parasite genetic factors responsible for the differential outcome of PKDL from South Asia versus East Africa?
• Is it possible to generate an animal model for PKDL using the existing knowledge of genetic susceptibility and altered immune pathology?

## Disclaimer statement

The funders had no involvement in the preparation of this manuscript, the decision to submit or in the design of any of the studies from the authors’ laboratories.

## Glossary

Amastigote antigen 2 (A2): an amastigote stage specific protein of *Leishmania* that plays a role in its survival within the mammalian host. It is induced by stress, and following heat shock it complexes with endoplasmic reticulum binding protein, BiP. By contrast, the A2 gene in *L. major* is a non-expressed pseudogene. A2 may also play a role in protecting *L. donovani* from stress associated with infection involving visceral organs, including fever typically associated with visceral leishmaniasis.
Amphotericin B: a polyene antifungal drug, often used intravenously for systemic fungal and parasitic infections including leishmaniasis; binds with ergosterol, a component of fungal and protozoal cell membranes, forms a transmembrane channel that leads to cell leakage and death.
Anthroponotic: an infectious disease caused by microorganisms that normally exist in a transmission cycle involving only humans, for example, tuberculosis and malaria.
CD25: the IL-2 receptor α chain present on activated T cells, B cells, natural killer (NK) cells, and monocytes. Additionally, CD25 along with FoxP3 is considered as one of the potent markers of T regulatory cells.
CD80 (or B7.1): is found on the surface of activated B cells and monocytes and provides the dominant co-stimulatory pathways that regulate T and B cell responses. It can bind to either CD28 to provide a stimulatory signal to T cells or can combine with cytotoxic T lymphocyte antigen 4 (CTLA-4) to send a negative signal to T cells.
CD86 (or B7.2): is a protein expressed on antigen-presenting cells that provide co-stimulatory signals necessary for T cell activation and survival.
Clinical cure: the disappearance of all clinical signs and symptoms, but does not necessarily mean complete elimination of the causative organism. On the contrary, sterile cure or radical cure is complete elimination of the causative organism. Occasionally, clinical cure is associated with the presence of the organism in a site other than the disease site, for example, leishmaniasis and malaria.
C-reactive protein (CRP): a protein found in blood, which increases in response to inflammation; it is considered as an acute-phase protein, that is, serum molecules that increase rapidly at the onset of infection.
Cutaneous leishmaniasis (CL): a tropical infection caused by vector-borne protozoa of the *Leishmania* species. It is often referred to as a group of diseases because of the varied spectrum of clinical manifestations, which range from small cutaneous nodules to gross mucosal tissue destruction. The disease is rarely fatal and can be subdivided into ‘self-healing’ or ‘non-self-healing’ forms.
Cutaneous T cell antigen (CLA): a unique skin-homing receptor expressed by memory T cells that infiltrate the skin; facilitates targeting of T cells to inflamed skin.
Cytochrome p450 27B1 (CYP27B1): a cytochrome P450 enzyme, also known as 5-hydroxyvitamin D_3_ 1-α-hydroxylase, located on the inner mitochondrial membrane. It catalyses hydroxylation of 25-hydroxyvitamin D_3_ at the 1-α position to synthesise 1-α, 25-dihydroxyvitamin D_3_, the active form of vitamin D_3_, which then binds to the vitamin D receptor and regulates calcium metabolism.
Dermatotropic: a higher affinity for the skin.
Epidermal Langerhans cells (E-LCs): Langerin (a Ca^2+^-dependent lectin receptor, CD207) positive dendritic cells that reside within the epidermis and act as major antigen-presenting cells during skin infections.
Ficolin-2: secreted pattern recognition lectins that contribute to the innate immune recognition of pathogens and activate the complement pathway to clear pathogens.
Forkhead box P3 (FoxP3): is also known as scurfin, and it is the master regulator as well as transcription factor of T regulatory cells. It inhibits the transcription of IFN-γ and activates transcription of IL-10.
Glutathione (GSH): a tripeptide (γ-glutamyl-cysteinyl-glycine) and the most abundant low molecular weight thiol in cells. It plays an important role in oxidation reduction reactions because GSH with glutathione peroxidase scavenges toxic hydrogen peroxide.
Glycoprotein 63 (gp63): a major surface glycoprotein of the genus *Leishmania* and is considered a major virulence factor of the parasite. Functionally, it is a surface Zn metalloprotease, and it has the ability to modulate macrophage signalling pathways.
Hapten-induced contact-dependent hypersensitivity: a Langerhans cell mediated CD8^+^ T cell dependent immune response elicited by epicutaneous sensitisation with haptens (i.e., chemicals including metals). Such reactions in mice and humans are mediated by Th1 or T cytotoxic 1 (Tc1) effector cells and downregulated by Th2 or T regulatory CD4^+^ T cells. The response develops in two distinct phases: (i) a sensitisation phase where the haptens penetrating the skin are captured by resident dendritic cells, which migrate to regional lymph nodes and induce activation of specific T cell precursors; (ii) the elicitation phase, which is induced by re-exposure to the same hapten at a remote skin site. Within a few hours, this leads to the rapid recruitment and activation of specific T cells and a local inflammatory response, which peaks at 24–48 h after challenge.
Haem oxygenase 1: an essential enzyme in haem catabolism that cleaves haem to form biliverdin and carbon monoxide. Haem oxygenase has two isoforms: haem oxygenase 1, which is inducible, whereas haem oxygenase 2 is constitutive in nature. Haem oxygenase-derived carbon monoxide and biliverdin have potent anti-inflammatory activity and can suppress macrophage function.
Human cathelicidin (hCAP-18): cathelicidins are a family of antimicrobial proteins among which hCAP-18 liberates the antibacterial, cytotoxic peptide LL-37.
Interferon-γ (IFN-γ): a cytokine produced primarily by T lymphocytes and NK cells. It induces the production of an array of cytokines and upregulates the expression of class I and class II MHC antigens, Fc receptor, and leukocyte adhesion molecules. It modulates macrophage effector functions, influences isotype switching, and potentiates the secretion of immunoglobulins by B cells. IFN-γ also augments helper T cell expansion and may be required for helper T cell differentiation. IFN-γR is expressed at moderate levels on virtually every cell with the exception of erythrocytes. Upon binding to IFN-γ, the receptor dimerises and activates transcription factors, resulting in increased production of proinflammatory molecules. Polymorphism in IFN-γR contributes to its lowered function and subsequently influences the cellular responsiveness towards IFN-γ.
Interleukin 10 (IL-10): an important immunoregulatory cytokine produced by many cell populations. Its main biological function seems to be the limitation and termination of inflammatory responses and the regulation of differentiation and proliferation of several immune cells such as T cells, B cells, NK cells, antigen-presenting cells, etc. Antigen-presenting cells and lymphocytes are the primary targets of IL-10 by its ability to regulate the Th1/Th2 balance. IL-10 promotes the development of a Th2 immune pattern by inhibiting the IFN-γ production of T lymphocytes, particularly via the suppression of IL-12 synthesis in macrophages. IL-10 is also secreted from T regulatory cells to counter balance inflammation.
Kala-azar: an alternative name for visceral leishmaniasis. The name originated from two words, kala (means black) and azar (means fever), attributed to the association of fever with hypopigmentation.
Keratinisation: a biochemical process of keratin deposition on cells. During this process, the epidermis sheds dead skin cells.
Major histocompatibility complex class II (MHCII) genes: MHCII genes encode peptide transporters expressed by antigen-presenting cells (macrophages, dendritic cells, B cells) that facilitate recognition of antigenic peptides by T cells; in humans, the MHCII genes are *HLA-DR*, *HLA-DP*, and *HLA-DQ*. Expression of MHCII genes is upregulated upon exposure to IFN-γ and inhibited by exposure to IL-10.
Mannose-binding lectin 2 (MBL-2): belongs to the collectin protein family and is an important element in the innate immune system. MBL-2 recognises mannose and N-acetylglucosamine on many microorganisms and is capable of activating the classical complement pathway.
Miltefosine (hexadecylphosphocholine): the first oral drug used for treatment of leishmaniasis. Miltefosine belongs to the class of alkylphosphocholine drugs, which are phosphocholine esters of aliphatic long-chain alcohols. The dosage for treatment of VL is based on the patient's body weight (b.w.): 100 mg/day (28 days) for b.w. >25 kg and 50 mg/day (28 days) for b.w. <25 kg. For the treatment of PKDL, the same dose has been used but the duration increased to 12 weeks. Miltefosine has a dual effect on leishmaniasis because it causes mitochondrial dysfunction, inhibition of cytochrome C oxidase, and increased generation of reactive oxygen species resulting in an apoptotic-like death in *Leishmania* parasites; in parallel, it raises the proinflammatory cytokines and molecules in *Leishmania*-infected macrophages and in patients with human leishmaniasis.
Papulonodules: a papule is a circumscribed, solid elevation of skin with no visible fluid and indurations, whereas nodules are larger solid areas of skin, with more than 0.5 cm area and indurations. Patients who have both papules and nodules are considered as papulonodules.
Parasite persistence: the presence of parasites even after clinical cure. It is generally seen in tryponosomasis and leishmaniasis.
Paromomycin: an aminoglycoside antibiotic isolated from *Streptomyces krestomuceticus* used for the treatment of VL.
Photoisomerisation: the light-initiated process of change from one isomeric form of a compound, radical, or ion to another. Importantly, in this process the changing in shape occurs without breakage of chemical bonding, an example being conversion of 11 *cis* retinal to conversion into all-*trans* by photons.
Post kala-azar dermal leishmaniasis (PKDL): a chronic, dermal sequel of VL or kala-azar that occurs in some but not all who are treated for kala-azar. PKDL is confined mainly to India and its adjoining countries, such as Bangladesh and Nepal, and it is also seen in Sudan and Kenya. Clinically, the disease is characterised by a macular, depigmented eruption found mainly on the face, arms, and upper part of the trunk.
Promastigote surface antigen 2 (PSA2): a surface glycoprotein of *Leishmania* expressed on both promastigotes and amastigotes. It has been proposed to be involved in host–parasite physical interactions. It plays a role in parasite attachment through complement receptor 3 (CR3) and is associated with the resistance against complement-mediated lysis. PSA2 is an immunologically reactive protein that induces high levels of Th1 cytokines and is therefore considered as a vaccine candidate.
Self-limiting PKDL: in Sudan, spontaneous healing frequently occurs in patients with PKDL, and is considered as self-limiting PKDL. In these patients, the lesions regress spontaneously with or without leaving scars.
Sodium antimony gluconate (SAG): a pentavalent antimonial that was the drug of choice for treatment of kala-azar or VL. After being converted into the trivalent form, it enters into macrophages/parasites and shows a dual effect. It causes an apoptosis-like death in *Leishmania* parasites by generation of reactive oxygen species, depletion of thiols, modulation of energy metabolism, and by inhibition of DNA topoisomerases. Additionally in macrophages, it induces a generation of reactive oxygen and nitrogen species by modulation of mitogen-activated protein kinases. Initially, Sb(V) was used in a dose of 10 mg/kg for 6–10 days, but increasing unresponsiveness in India led to successive upward revisions, and currently the amount of drug being used is ten times more than in previous years. Due to irregularity of use and poor patient compliance, resistance against this drug has emerged. Increased presence of drug efflux pumps and raised levels of non-protein thiols are the major molecular mechanisms responsible for drug resistance in parasites. Owing to high resistance, the usage of SAG is now restricted.
Solute carrier family 11A1 (SLC11A1): encoded by the *SLC11A1* gene in humans and is expressed in monocytes, macrophages, and neutrophils. It is an integral phagolysosomal protein composed of 12 hydrophobic transmembrane domains. Functionally, it acts as a divalent transition metal (iron and manganese) transporter on the endosomal membrane and is involved in iron metabolism and host resistance to certain pathogens. It was previously referred to as natural resistance-associated macrophage protein 1 (NRAMP1).
Sterile cure: (radical cure) the complete or 100% elimination of the pathogen from the host or organism. In a parasitic disease, sterile cure never happens.
Systemic memory response: attributable to the immune system mediated by circulating memory T or B cells, whereby a second encounter with an antigen induces a heightened state of immunity.
THP1 macrophage: a monocyte cell line derived from a patient diagnosed with acute monocytic leukaemia. This cell line is grown in suspension and is a non-adhesive type. Treating this cell line with PMA helps the cells to adhere and differentiate into macrophages.
Tissue-specific immunity: the immune response specific to the tissue site. This response is specific towards a specific antigen and is regulated by dendritic cells, macrophages, T cells, and B cells.
T regulatory cells (Tregs): are a proportion of T cells usually identified by the expression of the transcription factor FoxP3 and a high level of expression of CD25. They can be either CD4^+^ or CD8^+^ and are important in controlling heightened immune activation or inflammation. They act either through cell–cell interaction such as cytotoxic T lymphocyte antigen 4 (CTLA-4) or by the release of immunoregulatory cytokines such as IL-10 and TGF-β.
Tumour necrosis factor α (TNF-α): a strong mediator of inflammation, secreted primarily from monocytes, macrophages, activated T and NK cells. Through TNF receptors, it promotes cytotoxicity, induces angiogenesis, bone resorption, and thrombotic processes.
Urocanic acid: an intermediate of histidine catabolism with two isoforms of which the *trans* form is the naturally occurring form. *Trans*-urocanic acid is isomerised into the *cis* form by UVB light. *Trans*-urocanic acid acts as a natural sunscreen, providing a low-level protection against UVB-induced DNA damage. *Cis*-urocanic acid is proposed to have immunosuppressive properties because it activates T regulatory cells, decreases the density of Langerhans cells, and alters their morphology. In addition to its well-reported immunosuppressive properties, it can also initiate production of reactive oxygen species and cause oxidative DNA damage.
Visceral leishmaniasis (VL) or kala-azar: is an infection of the reticuloendothelial system, usually caused by the parasites *L. donovani*, *Leishmania infantum*, or *Leishmania chagasi*. The majority of VL cases occur in Bangladesh, Brazil, India, Nepal, and Sudan. It is the most fatal form of the leishmaniases if left untreated. The patient presents with fever, malaise, and weight loss associated with anaemia, hepatosplenomegaly, hypergammaglobulinemia, and concomitant hypoalbuminemia.
Viscerotropic: the affinity or tendency of an organism to reside within viscera.
Vitamin D receptor (VDR): a nuclear receptor, which upon activation by dihydroxylated vitamin D_3_, forms a heterodimer with retinoid X receptor (RXR) and causes upregulation of arginase 1 and cathelicidin (LL-37), along with subsequent downregulation of proinflammatory genes.
Vitamin D response elements (VDREs): a DNA sequence found in the promoter region of vitamin D regulated genes. It is present in many cell types including monocytes, macrophages, and dendritic cells. VDR, upon binding to dihydroxylated vitamin D_3_, enters into the nucleus and subsequently binds with RXR; the resultant complex binds to VDREs to regulate gene expression, of which the most potent is cathelicidin or LL-37.
